# A Case of Bilateral Macular Phototoxicity and the Role of Multimodal Imaging

**DOI:** 10.7759/cureus.99791

**Published:** 2025-12-21

**Authors:** Abraham Gabriel, Raef S Dimitry, Michael Milad, Monica Kelada, Katia Papastavrou

**Affiliations:** 1 Medicine, Ashford and St. Peter's Hospitals NHS Foundation Trust, Chertsey, GBR; 2 Ophthalmology, Frimley Health NHS Foundation Trust, Camberley, GBR; 3 Oncology, West Hertfordshire Teaching Hospitals NHS Trust, Watford, GBR; 4 Medicine, Imperial College Healthcare NHS Trust, London, GBR; 5 Ophthalmology, Pantheo Eye Center, Limassol, CYP

**Keywords:** general ophthalmology, macula, macula edema, prevention ophthalmology, retina, solar exposure, solar retinopathy, sunlight, sunlight exposure

## Abstract

Macular phototoxicity is a rare form of retinal injury caused by intense light exposure, most commonly from direct sun-gazing or viewing a solar eclipse without adequate eye protection. We report a case of bilateral macular phototoxicity in a 35-year-old female photographer with no significant past medical history, who developed acute central vision loss after approximately one hour of inadvertent sun exposure. She presented 48 hours after the incident with markedly reduced central vision (only light perception centrally) in both eyes, while peripheral vision remained intact. Dilated fundus examination revealed bilateral circumscribed focal foveal hypopigmentation in the absence of other retinal or optic nerve findings. Optical coherence tomography (OCT) scans confirmed foveal phototoxic damage, showing a normal overall contour but with juxtafoveal microcystic changes and hyperreflective lesions at the fovea in both eyes, consistent with acute macular phototoxicity. Conservative management with lubricating eye drops and oral nutritional supplements was implemented. Over the ensuing weeks, her visual acuity improved from an initial profound central scotoma to 20/40 in both eyes at 10 days, and 20/40 (left eye) and 20/30 (right eye) by one month post-exposure. Follow-up OCT demonstrated resolution of macular swelling and reconstitution of retinal layers. By five months, her visual acuity had fully recovered to baseline (20/20); however, a small persistent central scotoma remained in the left eye. This case illustrates the clinical course of macular phototoxicity and highlights the importance of patient education on ocular sun safety. Early recognition is important, as most cases show significant spontaneous recovery, but preventative measures are paramount since treatment options are limited.

## Introduction

Macular phototoxicity refers to retinal damage caused by exposure to intense solar or other high-intensity light radiation, exceeding the retina’s natural protective mechanisms [1]. Although classically associated with intentional sun-gazing or viewing solar eclipses without proper eye protection, macular phototoxicity can also occur from unintentional sun exposure during outdoor activities [2]. The pathophysiology primarily involves photochemical injury rather than direct thermal burn [3]. Ultraviolet-A (UV-A) light (315-400 nm) makes up ~95% of UV radiation reaching the earth’s surface and is chiefly responsible for retinal injury in these cases [4]. UV-A photons penetrate the eye (since shorter UV-B/C wavelengths are absorbed by the cornea and lens) and induce Type 2 photochemical reactions, generating reactive oxygen species that damage the photoreceptors and retinal pigment epithelium (RPE) [5,6]. Histologically, the damage is most pronounced at the fovea and in the parafoveal region, where cone photoreceptors (more sensitive than rods to UV injury) suffer oxidative stress, RPE cell disruption, and formation of necrotic lesions [7]. Clinically, patients with macular phototoxicity typically present within hours to days of the inciting light exposure with symptoms such as reduced central vision, central or paracentral scotomas, chromatopsia (colour distortion), metamorphopsia (visual distortion), and photophobia [8]. The condition often affects both eyes because the activity leading to exposure (e.g., sun-gazing or eclipse viewing) is usually binocular [9].

While many cases of macular phototoxicity are mild and resolve spontaneously over weeks to months, the degree of retinal damage can vary. There is no definitive treatment, though various interventions (high-dose corticosteroids, antioxidants, photodynamic therapy, etc.) have been attempted in severe cases with mixed results [10]. We present a case of bilateral macular phototoxicity in a patient without deliberate sun-gazing, illustrating the clinical course with multimodal imaging and discussing management considerations and prevention strategies.

## Case presentation

Patient information

A 35-year-old woman with no significant medical history presented to our ophthalmology clinic in July 2023 with acute, painless vision changes. She was a professional photographer and had been outdoors two days prior, spending approximately one hour lying face-up under a tree in the midday Cyprus sun without sunglasses or spectacles. She claimed not to have stared directly at the sun, and later that day, noted central visual blur in both eyes. By the time of presentation 48 hours later, she reported that her central vision was severely reduced, describing that she could only perceive bright light in the center of her visual field, while her peripheral vision remained intact. She also endorsed dryness and grittiness in both eyes. There was no history of recent illness, ocular trauma, or use of photosensitizing drugs. Her ophthalmic history included only mild astigmatism with hyperopia corrected by glasses, and physiologic optic disc asymmetry noted in childhood. She had a remote history of allergic conjunctivitis as a teenager. Family history was unremarkable. She had no history of diabetes, hypertension, or other systemic illnesses.

Clinical findings and diagnostic assessment

**Figure 1:**
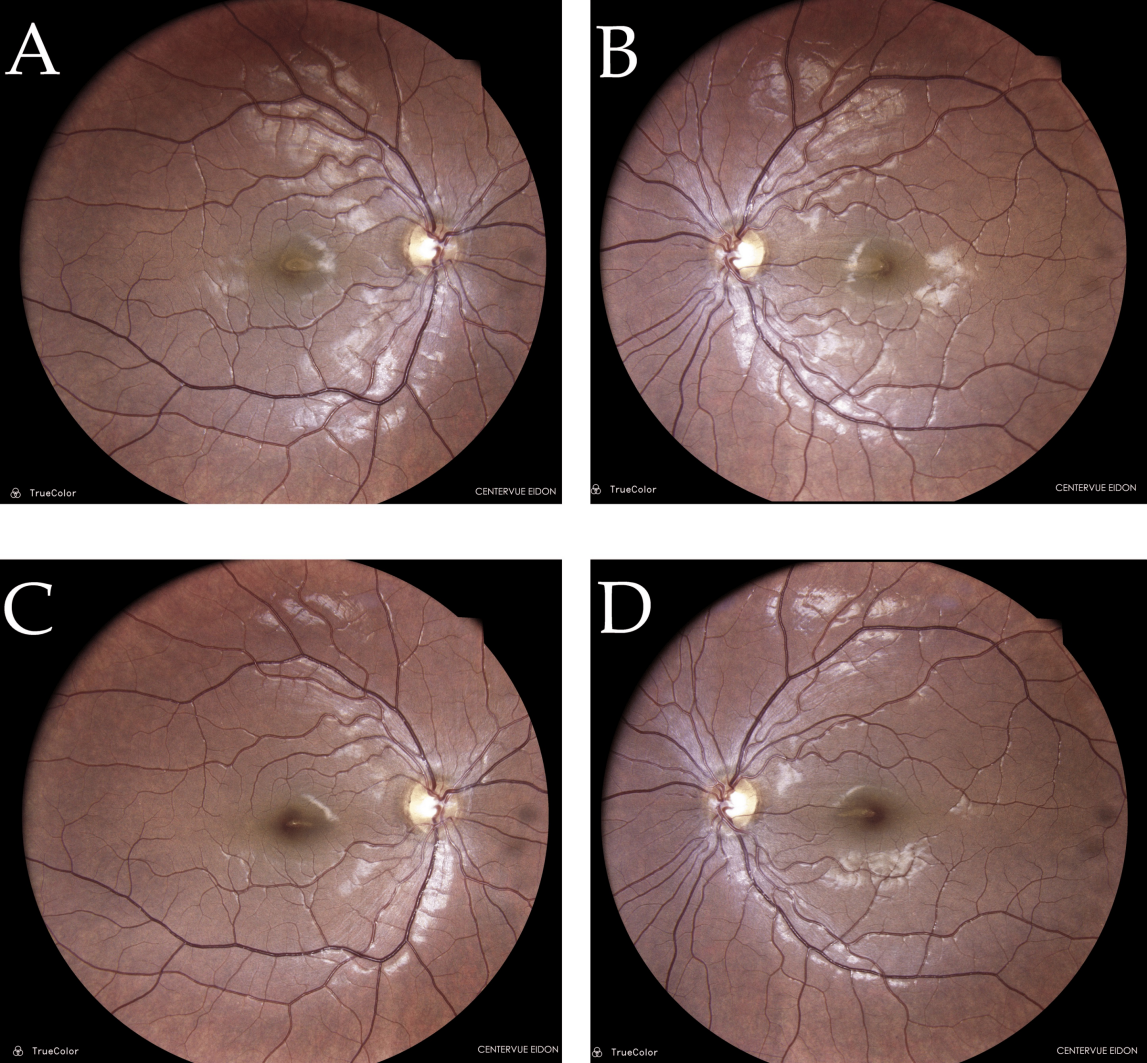
Baseline (A, B) and follow-up fundus photographs (C, D) of both eyes TrueColor fundus images were captured with the CenterVue EIDON system (CenterVue, Padova, Italy). Baseline photographs (A-B, Day 2 post-exposure) show a mildly hazy macular region with subtle central foveal yellowing and perilesional retinal whitening in both eyes. The right eye demonstrates a distinct parafoveal ring of mottled hypopigmentation, while the left eye shows a sharply defined yellow foveal lesion. Retinal vasculature and optic discs appear normal in both eyes. These findings are consistent with early phototoxic maculopathy, likely due to outer retinal and retinal pigment epithelium (RPE) disruption. Follow-up images (right fundus, C; left fundus, D) at one month post-exposure show significant resolution of the macular changes. The right and left maculae both appear less edematous, and the retinal pigmentary disturbances have faded, with restoration of more uniform macular pigmentation. No new lesions or hemorrhages are noted. This supports recovery from acute photic injury with partial RPE and photoreceptor integrity restoration.

On examination, her visual acuity at presentation was significantly reduced centrally (exact Snellen acuity could not be measured due to bilateral central scotomata, but she was unable to read standard charts, corresponding to only light perception in the central field). Microperimetry confirmed severe macular dysfunction, with a mean sensitivity of 16.2 dB in the right eye and 18.4 dB in the left eye. The right eye demonstrated a central absolute scotoma and unstable fixation, while the left eye showed a central relative scotoma with borderline fixation stability. Pupils were equal and reactive, with no afferent pupillary defect. Intraocular pressures were 14 mmHg and 15 mmHg in the right and left eyes, respectively. Anterior segment examination was unremarkable aside from mild conjunctival dryness bilaterally. Slit-lamp biomicroscopy with a 90D lens revealed healthy optic nerves and peripheral retina in both eyes (Figure 1). However, both maculae showed loss of the normal foveal light reflex and subtle retinal edema in the foveal region. There were no visible retinal hemorrhages, choroidal ruptures, or any retinal breaks/detachments; the retinas were flat and intact bilaterally. Color vision was full (Ishihara result, 17/17 each eye), indicating no permanent loss of cone function for color discrimination. Several diagnoses could be considered, including bilateral paracentral acute middle maculopathy (PAMM), acute idiopathic maculopathy, and occult macular dystrophies. However, given the history and exam findings, a provisional diagnosis of macular phototoxicity was made.

**Figure 2:**
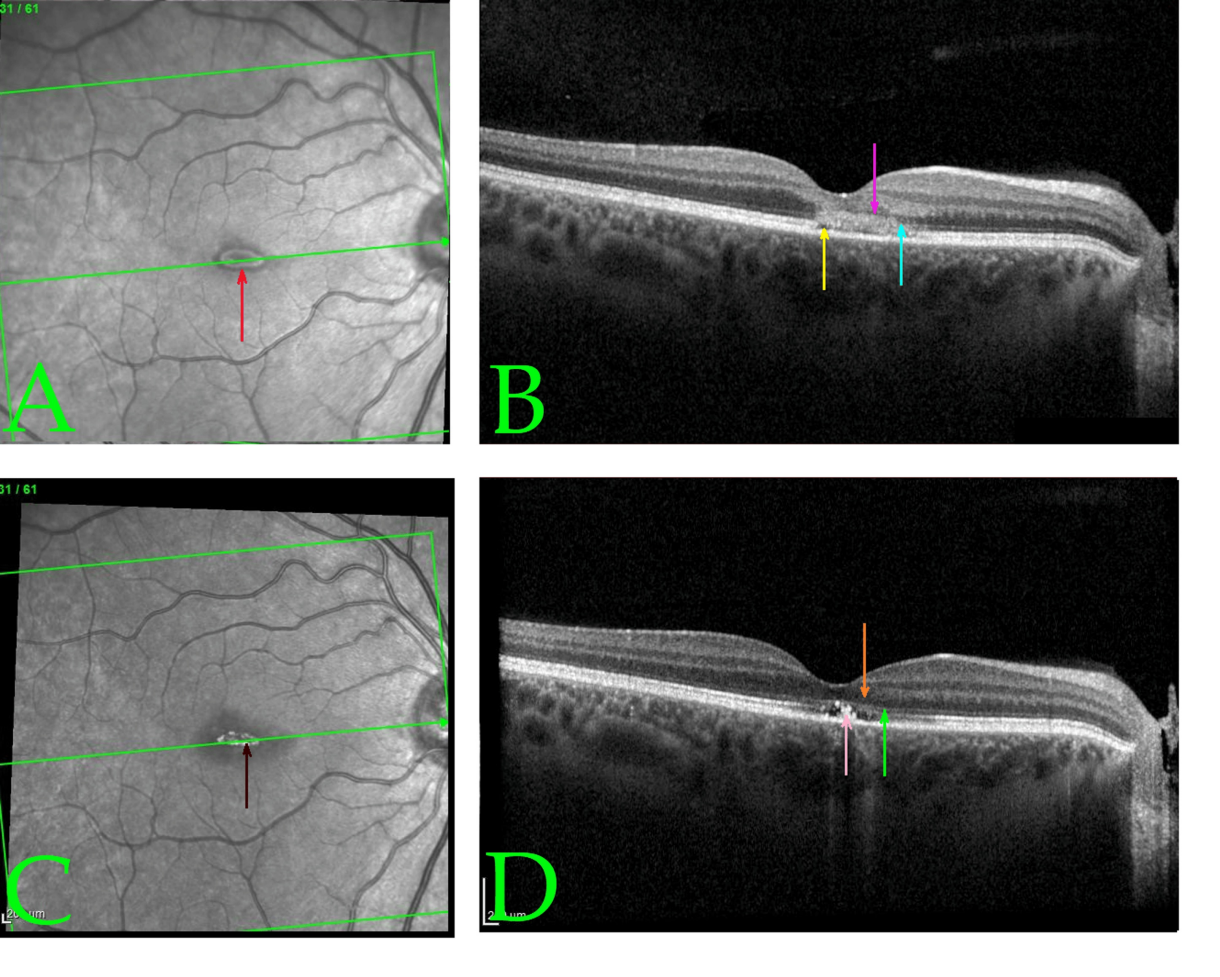
Infrared reflectance and optical coherence tomography (OCT) scans at baseline (A, B) and early follow-up (C, D) of the right eye demonstrating structural changes associated with acute macular phototoxicity and subsequent recovery Baseline infrared reflectance (A) and cross-sectional OCT (B) of the right eye, on Day 2 post-exposure (using Heidelberg SPECTRALIS®; Heidelberg, Germany), show a normal foveal depression but with signs of phototoxic damage. Both images were captured using Heidelberg SPECTRALIS OCT. In (A), the red arrow indicates a central foveal hyporeflective area suggestive of acute phototoxic injury. In (B), the yellow arrow identifies foveal outer retina disruption with intact retinal pigment epithelium (RPE). The pink arrow highlights disruption and hyperreflectivity in the outer nuclear layer at the fovea. The light blue arrow points toward focal hyperreflectivity with loss of the normally distinct ellipsoid and interdigitation zones at the photoreceptor–RPE interface. No subretinal fluid or macular hole is observed. Follow-up reflectance (C) and OCT (D), on Day 10 post-exposure, demonstrate early structural recovery. In (C), the brown arrow shows a central hyperreflective spot likely due to post-injury retinal tissue remodeling. In (D), the green arrow denotes subtle residual irregularity in the ellipsoid zone at the fovea. The orange arrow shows restoration of the outer nuclear layer, with reconstitution of the normal retinal lamination. The peach arrow marks persistent irregularity at the photoreceptor–RPE interface, which remained more apparent in the right eye. Central retinal thickness has reduced, indicating resolution of edema. These anatomical improvements correlate with visual recovery to 20/30 in the right eye.

**Figure 3:**
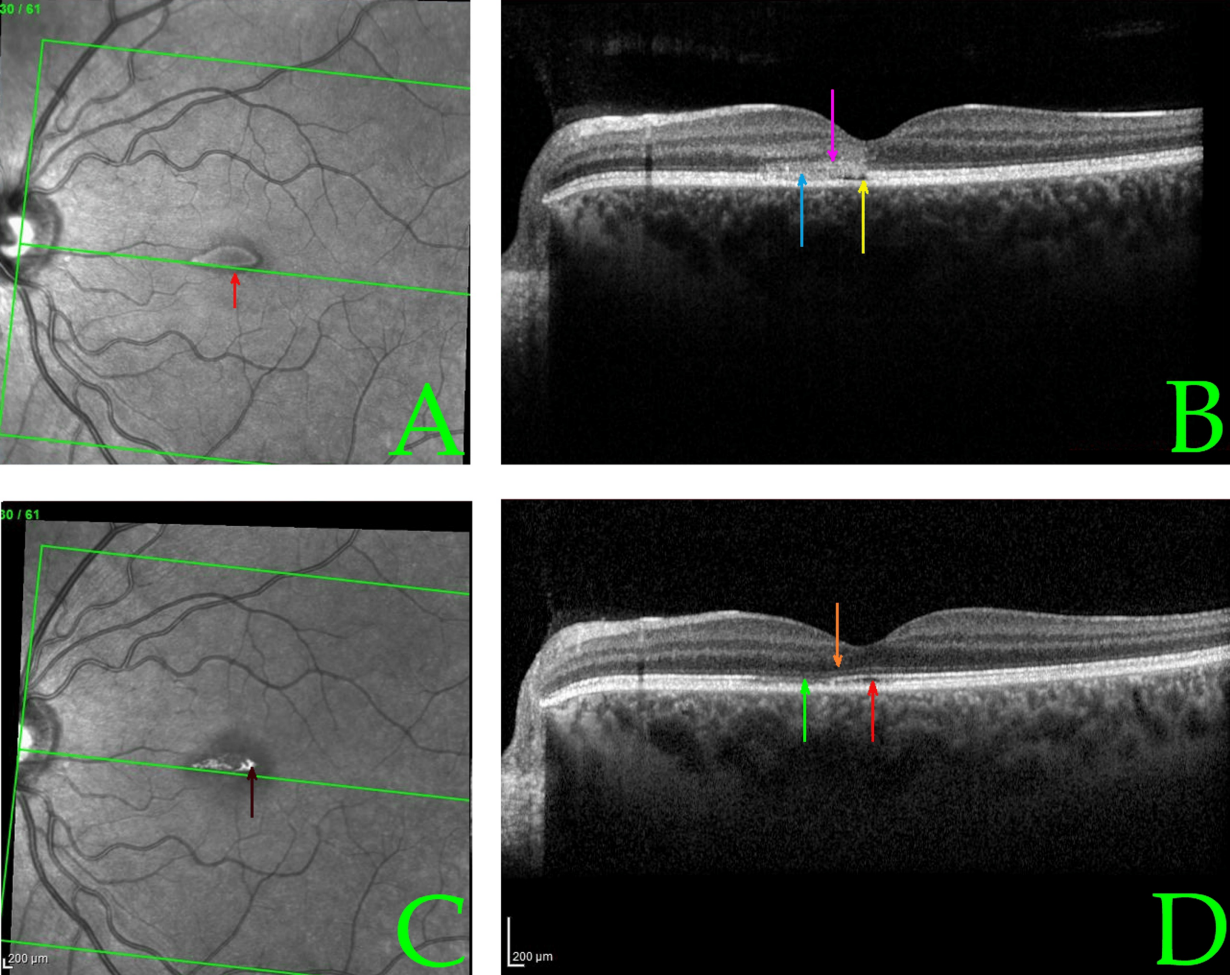
Infrared reflectance and optical coherence tomography (OCT) imaging of the left eye at baseline (A, B) and early follow-up (C, D), illustrating the structural evolution of acute phototoxic maculopathy At baseline (Day 2 post-exposure), (A) SPECTRALIS® infrared reflectance shows a central hyporeflective foveal lesion (red arrow), suggestive of acute phototoxic damage and (B) cross-sectional OCT reveals several acute injury features. The yellow arrow shows juxtafoveal hyporeflective microcysts, indicating intraretinal edema. The pink arrow indicates hyperreflectivity and disruption of the outer nuclear layer. The light blue arrow shows disorganization of the ellipsoid zone and photoreceptor–retinal pigment epithelium (RPE) interface. These findings are consistent with acute phototoxic maculopathy. No subretinal fluid or macular hole is observed. In Day 10 post-exposure (early follow-up) images, (C) infrared reflectance displays a central hyperreflective zone (brown arrow), likely representing tissue remodeling after injury. (D) OCT shows progressive structural recovery. The green arrow shows subtle residual irregularity in the ellipsoid zone. The orange arrow shows restored outer nuclear layer integrity and reformation of the normal foveal contour. The red arrow shows persistent irregularity at the photoreceptor–RPE interface, more apparent in the left eye at this time point. Central retinal thickness reduced from 180 to 168 µm, paralleling the resolution of outer retina lesions and visual improvement to 20/40 in the left eye.

Optical coherence tomography (OCT) was performed to confirm and assess the extent of macular injury. The initial spectral-domain OCT scans (obtained two days post-exposure) of both eyes demonstrated largely preserved overall retinal architecture with a normal foveal depression contour, but clear pathological changes at the fovea (Figures 2A, 2B, 3A, 3B). In both eyes, there were small, horizontal, band-shaped, hyperreflective lesions at the level of the outer retina and RPE, and foveal outer retina disruption with intact RPE. The ellipsoid zone (photoreceptor inner/outer segment junction) appeared blurred and disrupted in the foveal region, and the RPE-photoreceptor interdigitation line was indistinct -- findings consistent with acute photic injury [11]. No full-thickness macular holes or subretinal fluid were noted. Figure 3D shows the same OCT scan plane in the left eye at Day 10 post-exposure, illustrating the structural recovery described below.

Treatment

At the initial visit, treatment was started with frequent ocular surface lubrication (Euphrasia/hyaluronic acid eye drops, one drop in each eye three times daily) to address dry eye and support the ocular surface during recovery. The patient was counseled on strict avoidance of further bright light exposure without protection. Given the absence of any treatable macular tears or significant subretinal fluid, and the generally self-limited nature of macular phototoxicity, a conservative management approach was recommended for the retinal findings. Systemic or intraocular corticosteroids were not prescribed in this case, in line with a conservative management approach (the rationale being that no clear benefit of steroids in macular phototoxicity has been proven, and steroids carry risks such as precipitating central serous chorioretinopathy [12]).

Follow-up and outcomes

At the 10-day follow-up, the patient noted subjective improvement in her vision. Best-corrected visual acuity had improved to approximately 20/40 in both the left eye and right eye. The dry eye syndrome had continued to improve. The dilated exam at that time showed a reduction in macular edema and beginning return of the foveal reflex in each eye. A repeat OCT at 10 days post-exposure confirmed marked improvement: the previously seen microcystic cavities and hyperreflective lesions in the fovea were significantly reduced, and central retinal thickness had decreased when compared to baseline. The macular architecture was moving closer to normal. Given these improvements, the patient was advised to continue with observation. Oral multivitamin supplements (containing antioxidants such as vitamin C, vitamin E, lutein, and zeaxanthin) were recommended empirically to potentially aid retinal recovery, although evidence for their efficacy in this condition is limited [13].

By the third visit at 24 days (~3.5 weeks) post-exposure, the patient’s visual acuity had further improved to 20/40 (+) in the left eye and 20/30 (with pinhole) in the right eye. She reported only minimal persistent blurriness in the central vision of the left eye and nearly normal vision in the right. The ocular surface appeared healthy with resolution of punctate keratopathy, reflecting resolution of dye eye syndrome. The OCT at this visit was essentially unchanged from the 10-day OCT, showing a stable restoration of the foveal contour with no new findings. Figure 2 demonstrates the OCT findings in the right eye, analogous to those of the left. Figure 2B shows the initial right eye foveal OCT cross-section, and Figure 2D shows the follow-up at Day 10 post-exposure, mirroring the left eye’s recovery.

After the one-month follow-up, given the significant improvement and lack of active interventions beyond supportive care, the plan was to continue observation. The patient was advised to return for any new symptoms, and a final follow-up at around five months post-injury was scheduled. At that final visit (at around five months after exposure), her best-corrected visual acuity had returned to 20/20 in both eyes. She was symptom-free in the right eye. In the left eye, she noted a tiny persistent central dim spot (scotoma) in her vision, noticeable only when performing detailed photography work or reading fine print, which she learnt to cope with. Microperimetry at this stage confirmed near-complete functional recovery: the right eye demonstrated a mean sensitivity of 28.6 dB with no residual scotoma and stable fixation, while the left eye showed a slightly reduced mean sensitivity of 26.9 dB with stable fixation and a small persistent central relative scotoma, consistent with her symptoms. Dilated examination at that time was unremarkable, and OCT confirmed an intact retinal structure with only a faint trace of the prior foveal damage in the left eye (a slight irregularity at the photoreceptor inner-segment/outer-segment junction). Given the essentially complete anatomical and functional recovery, no further treatment was necessary. The patient, who was also a new mother, expressed that the restoration of her vision greatly improved her quality of life, although the slight residual left scotoma was an ongoing reminder of the event.

## Discussion

Our patient’s case illustrates the typical clinical course of macular phototoxicity, which often results in a spectrum of retinal changes that are largely transient but can lead to lasting deficits. Macular phototoxicity is fundamentally a photochemical injury to the retina, rather than a thermal burn [3]. Intense visible and UV light exposure triggers the formation of reactive oxygen species in retinal tissues, overwhelming antioxidant defenses and damaging cellular structures (particularly in the RPE and photoreceptor outer segments of the foveal region) [5]. In experimental and clinical observations of acute macular phototoxicity, retinal changes can be seen within 24-48 hours of exposure. A fundoscopic examination classically may reveal a small yellow-white or red spot at the fovea, often with a faint pigment halo, corresponding to RPE disruption and edema. In our patient, the acute fundus changes were subtle (loss of foveal reflex and slight edema), likely because the exposure was indirect (filtered through the tree canopy) and of moderate duration, resulting in a less pronounced lesion.

Multimodal imaging aids in diagnosing and understanding macular phototoxicity. Fundus autofluorescence (FAF) in acute cases often shows a well-demarcated hypoautofluorescent spot at the fovea (from RPE damage), encircled by a hyperautofluorescent ring due to surrounding lipofuscin accumulation in distressed RPE cells. (FAF was not performed in our case.) OCT, however, provided critical information. On spectral-domain OCT, acute macular phototoxicity is characterized by hyperreflective changes at the fovea spanning from the outer nuclear layer to the RPE, blurring of the ellipsoid zone, and occasionally small subfoveal cavities or cystoid spaces [11]. These findings were evident in our patient’s initial scans (Figures 1A, 2A). Over time, OCT changes tend to evolve into thinning or even a lamellar macular hole at the fovea as necrotic tissue is resorbed. In many cases (especially mild ones), the OCT results can return nearly to normal as the photoreceptor layers realign, as seen in our patient’s follow-up scans (Figure 3D). Notably, even when visual acuity recovers, some patients report persistent small scotomata due to tiny areas of non-functional retina at the very center of the fovea. This corresponds with our patient’s outcome: full acuity return with only a minute residual central scotoma in one eye.

The management of macular phototoxicity is largely observation and supportive care. There is no proven effective treatment to reverse retinal photochemical damage once it has occurred; fortunately, the prognosis in most cases is good with spontaneous improvement. Approximately 50%-83% of patients experience significant visual recovery over one to six months, often returning to baseline acuity. Our patient’s course was consistent with this, showing major improvement within the first month and full 6/6 acuity by five months. However, outcomes can vary. Factors associated with a worse prognosis include very prolonged direct sun exposure, the absence of any light-attenuating media in the eye (e.g., an aphakic eye with no crystalline lens will allow more UV to reach the retina), and lack of protective eyewear at the time of exposure. In our case, while the patient was not looking at the sun deliberately, being outdoors under intense midday sunlight for an extended period likely delivered enough scattered UV radiation to cause injury, possibly exacerbated by the high ambient UV index in Cyprus during summer [14].

Various treatments have been reported anecdotally or in small case series for severe macular phototoxicity, though none are established as standard of care. Treatments with high-dose corticosteroids (e.g., oral prednisolone ~1 mg/kg) administered soon after the injury have been attempted, aiming to reduce inflammation and secondary damage, with some reports of benefit [11]. However, steroids carry significant risks (such as precipitating central serous chorioretinopathy) and, in many cases, do not dramatically alter the course [15]. Accordingly, in our case, no steroids were used, nor were non-steroidal anti-inflammatory drugs (NSAIDs). Photodynamic therapy and focal laser photocoagulation have been tried in cases where macular phototoxicity is complicated by choroidal neovascularization or persistent subretinal fluid, again with mixed success and likely only helping in those specific complications. More recently, a case report described using a suprachoroidal triamcinolone acetonide injection in a teenage patient with macular phototoxicity, which was associated with anatomical improvement on OCT [16]. Antioxidant vitamins (such as those given empirically to our patient) are often recommended to mitigate oxidative stress, but there is no conclusive evidence in humans that they influence outcomes in macular phototoxicity [17]. Given their relative safety and the lack of documented adverse effects with their use in macular phototoxicity, our patient was prescribed a three-month course of multivitamins following initial presentation. Ultimately, the natural history in most cases is spontaneous partial or complete recovery as the retina heals over a few months [15]. During this period, management focuses on protecting the eyes from further damage (strict sun avoidance and use of sunglasses), maximizing remaining vision (temporary use of low-vision aids if needed), and treating any concurrent issues (e.g., dry eye syndrome).

In the age of social media, there have even been dangerous viral trends (videos encouraging practices like deliberate sun-gazing for supposed health benefits) that put uninformed individuals at risk [18]. Our patient’s profession as a photographer also raises the point that using cameras or optical instruments pointed toward the sun (even indirectly) can focus light into the eyes; photographers should be counseled on this hazard. Furthermore, certain populations require special caution: children and adolescents may inadvertently risk solar injury (for instance, due to viral social media challenges) [18]. Those with impaired judgment because of substance use or cognitive impairment are also more prone to stare at bright lights or the sun, and cases of photic maculopathy have been reported in such contexts [19]. Caretakers should be mindful of this. Early and repeated education can prevent cases of macular phototoxicity.

Prevention and public awareness

It is far better to prevent photic retinal injuries than to have to treat them. This case is a reminder of the paramount importance of prevention and patient education. Even brief unintentional exposure to intense sunlight can injure the retina. Patients often are not aware of the risk, especially outside of well-publicized events like solar eclipses. Patients should be counseled on proper eye protection during high-risk situations. For example, using certified solar eclipse glasses meeting the ISO 12312-2 standards during eclipse events, and wearing UV-400-rated sunglasses when spending prolonged periods outdoors are essential [20]. This is particularly important in high-risk environments with high surface reflectivity (snow, water, sand), low sun angles (e.g., near sunrise/sunset when people may gaze longer), and high atmospheric UV transmission at high altitudes. People in professions or activities that risk intense light exposure (e.g., astronomers, welders, photographers using telephoto lenses, pilots) must use appropriate protective eyewear.

Eye care professionals should actively educate the public, especially children and adolescents, about the dangers of even brief direct or indirect sun exposure to the eyes. It is important to debunk misinformation and warn younger individuals (and their parents) about permanent eye damage. Habits that protect against acute macular phototoxicity (like consistently wearing UV-blocking eyewear) carry additional long-term benefits by reducing cumulative UV damage. Such measures might lower the risk of other UV-related ocular conditions, including certain cataracts and possibly age-related macular degeneration, which share some pathogenic mechanisms with photochemical retinal injury over time. Healthcare providers can help mitigate the incidence of macular phototoxicity and related ocular injuries, and when cases do occur, prompt recognition and appropriate counseling on prognosis are important for patient management and reassurance.

In summary, the clinical course in this case, bilateral foveal injury with initial vision loss followed by substantial spontaneous recovery, is characteristic of macular phototoxicity. This case also highlights that any sufficient UV/visible light exposure, even without deliberate staring at the sun, can cause photochemical retinal injury. Fortunately, with supportive care and avoidance of further damage, our patient recovered very well. Ongoing public education and preventive measures are critical to reduce the incidence of this avoidable condition.

## Conclusions

Macular phototoxicity presents a diagnostic and therapeutic challenge due to its rarity and lack of specific treatment. In most instances, a good visual prognosis can be expected with conservative management, but even mild cases can lead to lasting visual disturbances such as small scotomata. This case underscores that prevention is paramount, as retinal damage from intense light exposure is largely irreversible.
